# Integrating wearable mobile health technologies into chronic heart failure management: Insights from a mixed-methods study and persona development

**DOI:** 10.1177/20552076251375967

**Published:** 2025-10-09

**Authors:** Laura Svensson, Carolin Anders, Christoph Dieterich, Oliver Heinze, Petra Knaup, Lina Weinert

**Affiliations:** 1 Institute of Medical Informatics, 9144 Heidelberg University, Heidelberg, Germany; 2 Klaus Tschira Institute for Integrative Computational Cardiology, Department of Internal Medicine III, 155992 Heidelberg University, German Center for Cardiovascular Research (DZHK), Heidelberg, Germany; 3 38948 RheinMain University of Applied Sciences, Wiesbaden, Germany; 4 Heidelberg Institute of Global Health, Section for Oral Health, 9144 Heidelberg University, Heidelberg, Germany

**Keywords:** Chronic heart failure, wearables, mobile health, qualitative methods, quality of life

## Abstract

**Background:**

Chronic heart failure (CHF) affects over 64 million people globally and often reduces quality of life (QoL), contributing to higher mortality. Wearable devices offer opportunities for continuous monitoring and self-management. However, patient characteristics and perceptions of wearables vary, and healthcare practitioners (HCPs) lack guidance on identifying patients who would benefit from such tools. This study investigates patients’ experiences with wearables for self-monitoring and develops personas to assist HCPs in tailoring CHF management.

**Methods:**

A mixed-methods approach was used, combining qualitative semi-structured interviews and quantitative QoL data via the Kansas City Cardiomyopathy Questionnaire (KCCQ-12). CHF patients received an Apple Watch and iPhone SE for tracking vital data and completing questionnaires and participated in semi-structured interviews. Descriptive analysis of KCCQ-12 scores and thematic analysis of interview transcripts informed the creation of patient personas based on previous findings.

**Results:**

Thematic analysis identified six main themes, including self-monitoring practices, barriers, and factors influencing acceptance. Most patients used wearables daily, reporting benefits like increased health awareness and improved communication with doctors. Barriers included technical issues and difficulty integrating study devices with personal ones. Quantitative analysis suggested a tendency toward higher QoL among interview participants. Four personas emerged, reflecting varying levels of motivation, literacy, and disease burden.

**Conclusion:**

Wearables show promise for improving CHF self-management by enhancing health awareness and providing reassurance. However, technical issues and integration challenges remain barriers. The developed personas offer HCPs a practical tool to personalize care and identify patients most likely to benefit from wearables.

## Introduction

Chronic heart failure (CHF) has been characterized as a condition causing symptoms such as dyspnoea, fatigue, and fluid retention, as the heart is unable to meet metabolic needs.^1–3^ Prevalence of CHF is on the rise, with 64 million affected people globally.^4^ This rise is attributed both to rising incidence of CHF in developing countries, increased survival rates, and an aging population in developed countries, as CHF is typically observed in older adults.^5^ Five-year-survival rates improved from 29% to 60% between 1970–1979 and 2000–2009, respectively.^6^

A recent systematic review and meta-analysis showed that patients with CHF commonly report moderate to poor Quality of Life (QoL) in the physical dimension.^7^ Moreover, patients with CHF report lower QoL or health-related Quality of Life (HR-QoL) than patients with other chronic illnesses.^8,9^ It has also been observed that lower QoL in patients with HF is associated with higher mortality.^10,11^ The 2022 American Heart Association guidelines^12^ now recommend using specific QoL measures to regularly screen for QoL in patients with HF. This recommendation aims to improve knowledge about symptom burden and the quality of treatment decisions.

### Self-management and support through mHealth

After CHF diagnosis, patients are suddenly confronted with the requirement to monitor their weight, intake of medications, lifestyle adjustments (e.g. dietary changes), and more regular doctor's appointments, disrupting their previous way of life.^13,14,15^

In order to offer more comprehensive ways to support these requirements, different tools, such as virtual visits and disease-specific mobile health applications (mHealth apps) have been developed. Feasibility and effectiveness of virtual visits have already been evaluated, showing higher patient satisfaction with virtual visits than with in-person visits for follow-up of cardiac implantable electronic device recipients.^15^ In a review published in 2022, Cruz-Ramos et al.^16^ identified ten mHealth apps designed for patients with CHF. Most common functionalities were the generation and presentation of medical recommendations and notifications (e.g. medication reminders). Besides these general functionalities, a third of the studied apps were able to monitor physiological patient data, such as physical activity and heart rate. Only one study used a wrist-worn wearable to collect this data.^17^

Yet, using a wrist-worn wearable such as a smartwatch in addition to an app for monitoring offers different opportunities: When worn, it allows for continuous, non-intrusive monitoring throughout the day and can provide enhanced personal health data. Simultaneously, these devices are deemed acceptable, as previous studies in patients with CHF showed high adherence to wearing smartwatches^18^ and high usability of the studied devices.^19^

Regarding patients’ handling of their own health data and interactions with wearables, research has produced conflicting results. While some studies identified that constant self-monitoring can increase anxiety and stress,^20^ other research has shown that wearable use can foster a feeling of safety instead.^20,21^ The possibility to access personal health data and to observe physical reactions to specific health behaviors (e.g. taking medication or not) can give patients a renewed sense of control of their condition.^20^ In addition, patients using self-monitoring tools reported feeling more involved in managing their condition and felt better informed to make decisions.^22^ Older findings also showed that self-monitoring itself has a positive effect on QoL of CHF patients,^23^ suggesting a high usefulness of combining the concept and necessity of self-monitoring with the provided support of a wearable tool.

### Adoption and use of wearables

Although it is a commonly held belief that older patients are less likely to both use and benefit from technologies such as wearables, research on this topic has led to differing results. In 2022, Paolillo et al.^24^ conducted an observational study with patients with CHF using wearables. The results showed that older adults in their study had a higher adherence to the study protocol. Still, older adults have specific challenges in using technology, such as less experience and manual difficulties.^25,26^ Hence, from the perspective of healthcare practitioners (HCPs), it can be difficult to anticipate whether an individual CHF patient will benefit from a wearable for self-monitoring, as opposed to more traditional methods such as a blood pressure cuff and paper-based diary entries. Practical guidance to support HCP in their decision-making is missing.

### Study context

In an effort to (a) study patient experiences with using a wearable for CHF management and (b) develop practical decision-support assistance for HCP, the study presented in this paper used a wrist-worn wearable with an accompanying app to collect data from patients. Heart rate and physical activity were recorded via the wearable's sensors. Additional data included patient-reported outcome measures, such as QoL, as well as blood pressure and weight. To record and store patient reported data, this study used the phellow app, which was initially developed at Heidelberg University Hospital.^27^ The app provides different functions for patients in this study, for example, access to their medical record, filling in QoL questionnaires, and viewing appointments. An earlier study undermined the value and good usability of the phellow app for patients.^28^

Study participants of the study were recruited from the HiGHmed initiative,^29^ supported by the joint Center for Innovative Care (Zentrum für Innovative Versorgung, ZIV), a network of five university hospitals in Baden-Württemberg, Germany, which aims to promote the use of innovative digital applications for patients.

### Study objectives

This study aims to gain insights into patients’ attitudes and beliefs towards using a wearable and an app for self-monitoring. As a final step, development of personas, representing common types of CHF patients, their disease burden, and willingness to use wearables, is expected to inform and support HCPs in anticipating which patient would potentially benefit from the use of a wearable to support self-management. Personas have been used in various studies investigating the implementation of mHealth applications.^30–32^

## Methods

### Study design

The following study collected quantitative and qualitative data to provide a rich base for data analysis. Combining these methods with findings from the literature allowed for the development of personas representing common types of CHF patients, their disease burden and willingness to use wearables. The study employed an explanatory sequential design.

### Procedure

#### Study population and recruitment

Potential study participants had to receive treatment for cardiomyopathy at the clinic for Cardiology, Angiology and Pneumology at Heidelberg University Hospital. Patient recruitment took place from January 2023 to June 2023. Further inclusion criteria were age >18 years and the ability to understand and consent to the study. Patients fulfilling these criteria were invited to participate in the study. All interested patients received written and verbal information regarding the content and aim of the study and the respective data protection regulations.

Participants then received an iPhone SE and an Apple Watch Series 6 in order to continuously measure vital signs (heart rate, activity data). Furthermore, patients were asked to document additional health data (blood pressure, weight) in a separate app. The app also allowed for the collection of QoL data through a disease-specific questionnaire.

On the informed consent form, patients could indicate whether they were interested in participating in additional interviews to study individual experiences with self-monitoring. Participants who consented to these interviews were contacted individually to schedule appointments for the interviews, following a convenience sampling strategy.

#### Instruments

The Kansas City Cardiomyopathy Questionnaire (KCCQ-12) in a validated, German translation was used as a specific measure for QoL. This questionnaire allows for the measurement of physical limitations, symptoms, self-efficacy, social interference, and QoL in patients with cardiomyopathy.^31^

In addition to this quantitative measure, semi-structured, open-ended qualitative interviews were conducted with patients to gain a deeper understanding of their experience with the self-directed collection of vital signs. The interview guide was developed and pre-tested by LS and LW following a cognitive pre-testing method.^32^ Afterwards, changes were made to improve clarity of the interview guide. The final, translated interview guide is provided in Appendix 1. The qualitative part of this study is reported according to the “Standards for reporting qualitative research” (SRQR) from O’Brien et al.^33^ For reporting of the mixed-methods approach, the “Mixed Methods Reporting in Rehabilitation & Health Sciences” (MMR-RHS) guideline by Tovin et al. was followed.^34^ The recruiting process was conducted following the qualitative approach of thematic and coding saturation,^35^ which was achieved during the interviews.

##### Data collection and analysis

Participants were asked to complete the KCCQ-12 at baseline, monthly in the first three months and afterwards once every three months through the provided app. Quantitative data collection for the purpose of this analysis took place from January 2023 to July 2024. Quantitative study data from the standardized KCCQ-12 were provided for analysis by the Medical Data Integration Center of Heidelberg University Hospital. Descriptive analysis of the quantitative data was performed with SPSS (Version 22.0) by LS. The analysis included the calculation of a monthly as well as an overall KCCQ-12 score, reflecting both the development of QoL over time and the overall score as an objective measure of burden of disease. The frequency of questionnaire completion by individual participants was analyzed to draw conclusions about adherence to this mode of questionnaire collection.

After approximately three months of smartwatch use, participants were interviewed regarding their experiences with using the digital tool. Interviews took place either via phone or face-to-face and were conducted by LS, who is a health services researcher trained in qualitative interviewing. Interviews were recorded and transcribed verbatim. After transcription, LS and LW familiarized themselves with the data by reading and discussing the transcripts. Then, thematic analysis^35^ was used to code the transcripts, which allowed for both inductive and deductive coding. MAXQDA Standard 2020 (version 20.4.1; VERBI GmbH) was used to support this step. Both researchers coded all the transcripts independently and compared their results. Through a focused discussion, a final coding scheme was developed (Appendix 2).

**Figure 1.**
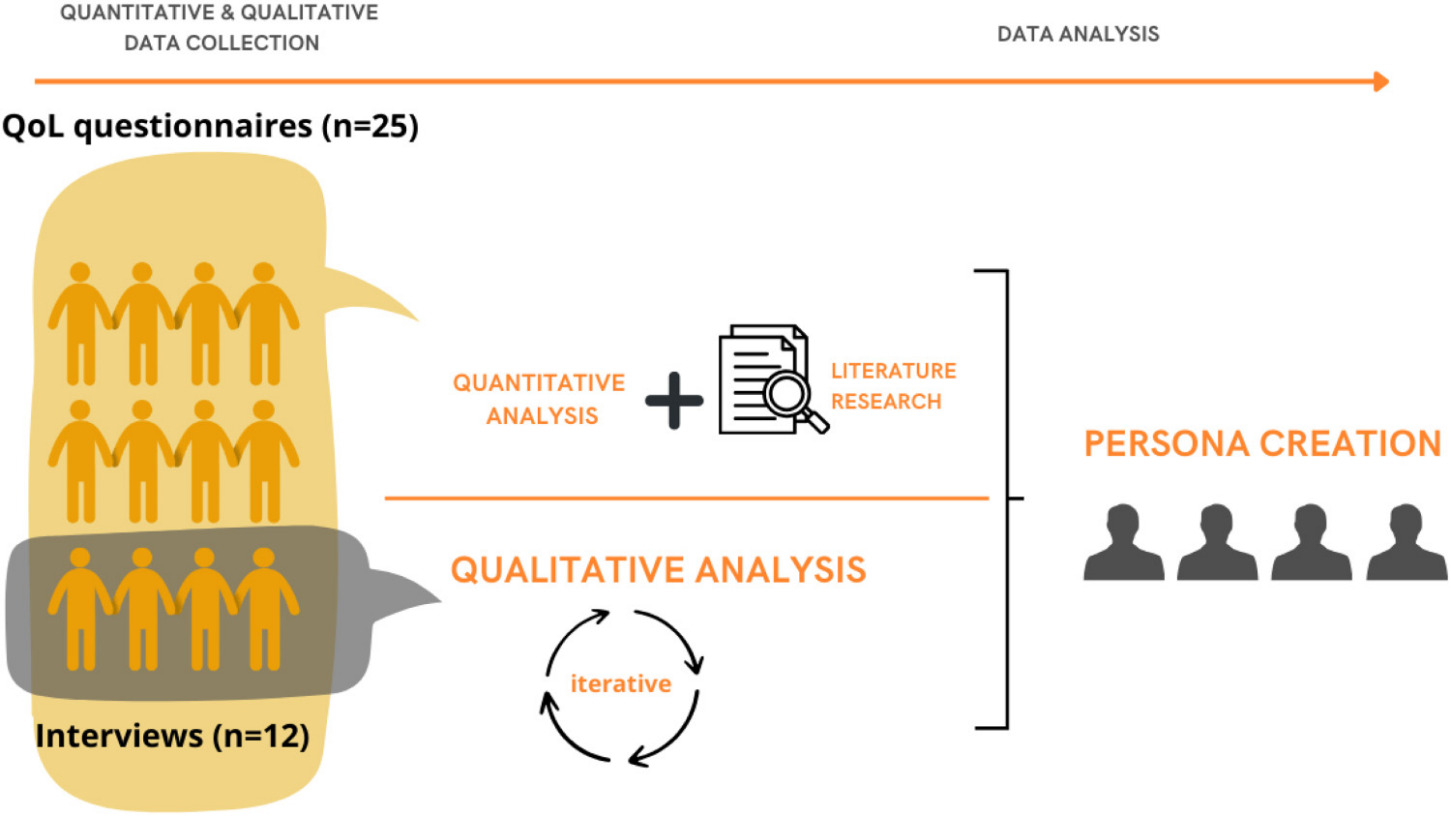
Visual depiction of data collection & analysis procedures.

Figure 1 visualizes data collection and analysis procedures.

#### Persona development

Based on interview findings, analysis of the quantitative data and sociodemographic results, three personas were developed. The categories were established inductively by screening the coding system derived from the qualitative analysis. This described creation process was based on George Olsen's Persona Creation and Usage Toolkit^36^ and leverages the integration of qualitative and quantitative findings in the chosen mixed-methods design.

An additional persona, representing a non-user, was created using information from the literature focused on reasons of patients not to use mHealth applications as a supporting tool for health management.

#### Ethical considerations

The study was conducted in accordance with the Declaration of Helsinki and approved by the Ethics Committee of Heidelberg University Hospital (S-966/2020). All participants provided written informed consent. Confidentiality and anonymity were ensured throughout the study. Data was protected against unauthorized access. No incentives or compensation was provided to participants for study participation.

## Results

### Interviews

Twelve out of 23 study participants were willing to be interviewed. The mean duration of the interviews was 21.5 min. Four interviews were conducted in person at the clinic and 8 via telephone.

Sociodemographic data of interview and non-interviewed participants are shown in Table 1.

**Table 1.** Sociodemographic data of interview and non-interviewed participants.

Gender	
*Interview participants*	
Female	8 (66.7)
Male	4 (33.3)
*Non-interviewed participants*	
Female	5 (45.5)
Male	6 (54.6)
	
Age	
*Interview participants*	
30–40	2 (16.7)
41–50	3 (25)
51–60	7 (58.3)
	
*Non-interviewed participants*	
30–40	4 (36.4)
41–50	4 (36.4)
51–60	3 (27.3)
	
Education	
*Interview participants*	
Academic degree	1 (8.3)
High school education	2 (16.7)
Lower or intermediate secondary education	9 (75)
	
Employment	
*Interview participants*	
employed	9 (75)
unemployed	1 (8.3)
retired	2 (16.7)

In total, 424 text passages were coded in the interview transcripts. A total of six themes and 73 subthemes were discovered. The following themes were identified:

- Self-monitoring
- Expectations
- Suggestions for improvement / factors to increase acceptance
- Motivation to participate in the study
- Barriers
- Acceptance/contributing factors

#### Self-monitoring

Various questions in the interview were aiming to explore how self-monitoring with the wearable was experienced by the interviewees. The following subthemes were found to be addressed in the interviews: Usage patterns, benefits of self-monitoring, self-monitoring before the study, effects of self-monitoring, and dealing with deviating values.

#### Usage patterns

When asked how frequently the watch was used, most patients stated that they would check the watch multiple times a day. Moreover, the usage pattern was highly influenced by personal wellbeing: patients replied that they would check the watch more often when feeling unwell.

Actually, I look at them every day, so I always check how things have been today and, as I said, if I'm not feeling well or something, then I also look at how they are at the moment, the data. (Interview 8, transcript position 39)

#### Attitudes towards self-monitoring

Two dominating attitudes were described by the interviewees: Firstly, the watch was perceived as a beneficial tool to help monitor the health status.

However, some patients had a generally negative attitude towards self-monitoring since they did not like the feeling of being controlled by the watch and did not want to become too dependent on the watch.

I notice how my body makes me feel in the end and I don't stress myself with the vital signs, I don't want to put myself under pressure. (Interview 12, transcript position 7)

#### Benefits of self-monitoring

Patients named numerous factors that they perceived as beneficial when using the watch. They stated that the watch would give them more flexibility and would simplify disease management. The ability to compare personal health data was perceived as very useful.

Being able to get an overview over the health status was not only seen as helpful for self-management but also as helpful to facilitate and simplify contact with doctors.

I think it's actually quite good that it's also useful for you to reflect on it again how up-to-date your blood pressure is, for example, or to check all the data yourself. (Interview 5, transcript position 6)

For most patients, the increased sense of security was a key benefit of using the watch. Interviewees stated that they no longer had to solely rely on their instincts on how their health status currently was but were able to actually check it.

Even if I notice, oh I think I am trembling, it is not yet atrial fibrillation but better calm down now. I just see that and before I couldn't, so I feel safer. (Interview 6, transcript position 13)

#### Effects of self-monitoring

Patients were asked about the effects that self-monitoring had on their daily life.

Although some patients stated that using a watch had only little effect on their general situation, most interviewees replied that the watch influenced their day-to-day habits of self-monitoring a lot. It was pointed out that controlling the health status would happen much more frequently now than before using the wearable. Most patients checked their data on a daily basis.

I look at the data everyday, I always check how I have been today and, as I said, if I'm not feeling well or something, I also look at what the data says at the moment. (Interview 8, transcript position 39)

Patients also mentioned that they felt more aware of their health status during the usage of the wearable. They would for example actively check their personal health data when wearing the watch, mostly in situations when they were feeling unwell but some stated that they would check on a continuous basis.

I consciously look at them on days or after days when I've either done something particularly strenuous or something like that or when I realize I'm not feeling well. (Interview 6, transcript position 89)

Some interviewees also stated that being able to monitor their health independently would make them feel more responsible for their situation.

So, with this research it makes me more responsible in monitoring my blood pressure and everything. (Interview 1, transcript position 18)

Interviewees were asked how they would act when observing deviating values that are too high or too low. Some patients stated that they wouldn't pay too much attention to it, often due to the fact that they are already accustomed to it. However, most of the other interviewees would react with increasing worries, depending on the significance of the deviations. While some patients would still continue with their day-to-day business while being a bit more alert, others stated that they would often find themselves caught in worrisome thoughts and would feel very uneasy.

So and yes, I can't do anything then, so I'm very agitated inside, very restless, yes, maybe it's also too much imagination, what's going on there, yes, fears come up about what could be or why it's like this now […] (Interview 3, transcript position 78)

When discovering deviating values, patients reacted with different strategies.

Some—mostly those that did not worry too much in the first place—would continue without taking special actions. Others—especially those with more worries—would try to slow down when the blood pressure was significantly higher or lower than expected. However, most interviewees would watch the values in the device continuously to determine if they would normalize again or if further actions are needed.

I then simply try to avoid this strain or to do it in such a way that I can ultimately say I can manage it so that my pulse doesn't go up. At work, I just do the heavy work at a slower pace. I then adjust my pace and then I can keep my heart rate down. (Interview 12, transcript position 39)

However, besides different reactions none of the patients perceived the awareness of deviating values due to the watch as a burden. Most of the interviewees perceived it as an enrichment to have the possibility to prevent negative consequences.

#### Expectations

Patients were asked if they had certain expectations towards the watch and the app when deciding to participate in the study.

Some patients mentioned that they expected the watch to be of help when monitoring their health. Others however were worried that the daily supervision would cause stress.

I was afraid it would be a burden because I really thought at the beginning that I would be looking at it all the time and oh dear oh dear. (Interview 6, transcript position 99)

Data privacy was also mentioned as an issue: an interviewee worried that they were not able to oversee what would happen to their data when participating in the study.

#### Suggestions for improvement / factors to increase acceptance

This code summarizes suggestions patients in this sample made to improve the self-monitoring via watch and factors that were mentioned to increase its acceptance.

Interviewees expressed a wish for an extended range of functions. Popular functions were the visualization of the data, an included sphygmomanometer, a medication reminder and an alarm for anomalies.

Some patients suggested that monitoring of the collected data by health care providers would be beneficial.

Besides these suggestions, patients in this sample also voiced the desire to be able to use the watch and the smartphone for personal use as well. Some patients—especially those that already owned a smartwatch—were annoyed that they had to carry two sets of smartphones and watches to both keep using their own devices and to comply with the study procedures.

I asked in advance if the app could not be transferred to my private device because I am already equipped with an iPhone, I am equipped with an Apple Watch, so to do that twice was unfavorable. (Interview 2, transcript position 2)

#### Motivation to participate in the study

Motivations to participate in the study were diverse. A lot of patients in this sample named their interest in monitoring their own health as a main reason to participate.

Yes, because I had heart failure […] it was important for me to take a look at my values during exercise and also to see how high my heart rate is in different situations in order to find out where I could perhaps improve myself and where I might need to slow down a bit. (Interview 10, transcript position 9)

Many patients also stated that their motivation to participate was to help other patients by donating their data for research.

I can then help other people, so by taking part, it will help someone else at some point, so I think that's good too. (Interview 11, transcript position 61)

A general interest in supporting research and generating knowledge about cardiomyopathy was an important factor to participate in for a lot of patients as well.

[…] because there are many people who also have the disease and who can later benefit from the data in a way that I may not have now, but others will have success later and it will be easier and better to do something about it. (Interview 12, transcript position 55)

Moreover, for some patients the curiosity to use an innovative method to self-monitor health was a reason to participate. These patients stated that they felt curious about trying a new way to support their disease management.

[…] that helped me a lot to simply understand whether I need such a watch, do I not need it and how do I use it? (Interview 6, transcript position 5)

#### Barriers

Patients in this sample named a number of barriers that complicated their utilization of the wearable. Barriers can be sub-divided into the categories of system-level limitations, usability issues and study design related barriers.

#### System-level barriers

System-level barriers were mentioned most frequently. Among those, interviewees mostly complained about problems with data transfer. They stated that they weren't able to send the data to the clinic or received an error message that their health data was not properly recorded.

Yes, so at the beginning of the use I entered my data, clicked on send and then it said 35 vital data could not be transmitted every time, so I was a bit unsure whether anything was transmitted at all because the display was always stuck on “could not be transmitted. (Interview 10, transcript position 21)

Interviewees mentioned a couple of other system-level barriers, for example problems with updates and appointments that were not up to date.

Some patients in this sample also criticized that the data was not monitored by medical staff, which they perceived as having no direct positive impact of participating in the study.

#### Usability issues

Besides system-level barriers, patients stated that they had usability issues, mostly with implementing the watch in their day-to-day life. Some mentioned a lack of time to use the smartwatch as frequently as recommended. Others complained that they did not like the dependence on the watch or that they were forced to carry it around at all times.

Furthermore, patients perceived it as a barrier that they were not able to use the wearable and the accompanying smartphone for personal use. Some patients were already using similar devices and hence found it stressful to use two sets of devices at the same time.

Well, I was hoping that I would be able to utilize it more for personal use, but that's just not possible. (Interview 7, transcript position 15)

In addition, some older patients had difficulties using the smartwatch correctly. They mentioned that it took some time to get accustomed to it and they complained that no clear instructions on how to use the watch in general were given.

#### Acceptance/contributing factors

A number of factors were named by the interviewees that contributed to the acceptance of the wearable. A lot of patients felt motivated to use the watch because of its useful functions, like the blood oxygen, pulse measurement, or the ECG function. Functions that are not directly connected to disease management like the exercise function, the sleep monitoring or a mindfulness trainer were perceived as very motivating to use the watch. However, others complained about too many unnecessary functions.

For example, I always find this mindfulness exercise really exciting >laughs< […] at some point I just go like this, now breathe for a minute, and I think that's really great, where I think that's what I do all day, but then I'm really focussed and I'd like […] to do it more often. (Interview 7, transcript position 97)

Moreover, patients that were already versed in the use of smartphones and smartwatches were more likely to have a higher acceptance using the watch.

I have an Apple Watch and an iPhone myself and know my way around a bit and yes, it wasn't a problem. (Interview 5, transcript position 8)

For some patients, curiosity was a factor that made them use the watch: these patients stated that they felt excited about being able to monitor their health.

### Questionnaires

QoL was measured at baseline and monthly using KCCQ-12 through the provided app (Table 2). The questionnaire scores were calculated as described in Spertus et al.^31^ KCCQ-12 scores were scaled from 0 to 100 and categorized into four categories using cutting points every 25 points. That is to say, scores from 0 to 24 indicated very poor to poor QoL; 25 to 49 indicated poor to fair QoL, 50 to 74 indicated fair to good QoL, and 75 to 100 indicated good to excellent QoL. To analyze the benefits on QoL over time, changes of 5 points in the score were considered to be small but clinically important, changes of 10 were considered moderate to large, and changes of 20 were considered large-to-very large clinical changes.

**Table 2.** QoL analysis.

	Quality of Life KCCQ-12 scores			Number of completed questionnaires
	Baseline	Range [Max;Min]	Mean (SD)	
Interviewed participants				
1	59	2 [59;57]	57.75 (0.96)	4
2	62	1 [63;62]	62.88 (0.35)	8
3	53	11 [64;53]	61.75 (3.92)	8
4	43	13 [56;43]	50.38 (3.28)	13
5	62	6 [63;57]	61.6 (1.78)	10
6	27	6 [27;21]	23.88 (1.78)	16
7	49	2 [49;47]	48.17 (0.96)	6
8	47	15 [62;47]	57 (4.31)	15
9	29	14 [33;19]	27.25 (5.37)	8
10	56	8 [64;56]	62.75 (2.76)	8
11	41	16 [57;41]	53.5 (4.53)	16
12	56	-	56	1
Total group	Mean 48.67 (SD 11.83)	45 [64;19]	51.91 (13.2)	113
Non-interviewed participants				
1	39	23 [59;36]	48.29 (8.41)	14
2	34	9 [37;28]	32 (4.24)	4
3	51	7 [51;44]	47 (2.31)	7
4	47	-	47-	1
5	60	3 [63;60]	62.25 (1.5)	4
6	27	12 [31;19]	28.27 (2.94)	15
7	43	12 [43;31]	36.67 (6.03)	3
8	49	16 [57;41]	47.82 (3.66)	17
9	48	-	48-	1
10	33	-	33-	1
11	19	-	19-	1
12	52	- [52;52]	52 (0)	2
13	54	-	54-	1
Total group	Mean 42.77 (SD 11.76)	44 [63;19]	42.72 (12.02)	71
Total population	Mean 46.60 (SD 11.93)	45 [64;19]	47.13 (13.20)	184

**Figure 2.**
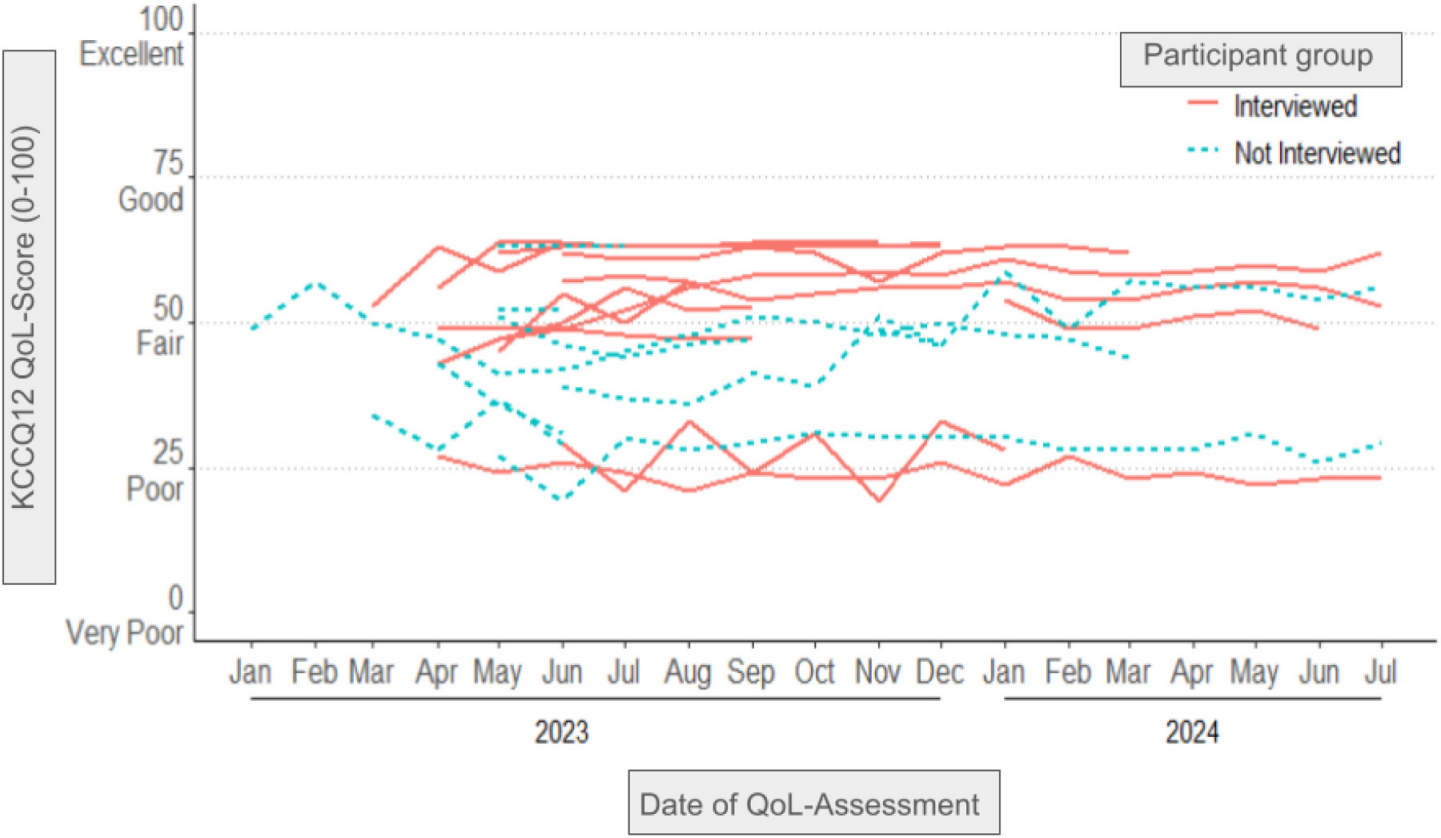
Progression of KCCQ-12 scores.

The progression of QoL of the participants over time is illustrated in Figure 2. Participants who were interviewed (orange lines) showed an overall higher QoL than those not interviewed (blue dotted lines). Most participants from the interviewed group had fair to good QoL (KCCQ-12 = 50 to 74). Most participants from the non-interviewed group had poor to fair QoL (KCCQ-12 = 25 to 49). However, in both groups, QoL seemed to remain unchanged over time. No participants registered good to excellent QoL (KCCQ-12 ≥ 75) and very few had very poor to poor QoL (KCCQ-12 ≤ 25). Subgroup analyses were performed in order to determine whether interviewed participants were representative for the whole group of study participants.

x = QoL-score.

y = point of inquiry: orange lines depict the progression of QoL-scores of participants that participated in an interview, blue dotted lines depict the progression for non-interview group's participants.

Overall mean KCCQ-12 QoL score in the study population was 47.13 (SD 13.20) [95% CI 41.68; 52.58]. The overall mean QoL of the group “interviewed participants” was 51.91 (SD 13.2) [95% CI 43.52; 60.3] and the overall mean QoL of the Group “non-interviewed participants” was QoL 42.72 (SD 12.02) [95% CI 35.45; 49.98]. T-test comparing baseline QoL group “interviewed participants” versus group “non-interviewed participants” resulted in *p*-value of 0.083, indicating no significant difference of QoL among the groups. However, due to the high percentage of missing data, the overall mean scores and the *p*-value are likely biased.

The average number of completed questionnaires per person for the total population was 7.36 (SD 5.66), for group “interviewed participants” 9.42 (SD 4.78) and for group “non-interviewed participants” 5.46 (SD 5.92). Among all participants in the study, the mean value of the number of completed questionnaires was seven, with five users having submitted one questionnaire and four users having submitted 15 or more questionnaires.

### Personas

Based on the interviews and a review of the literature, the following categories for persona characterization were established: motivation for participation, interest and competence regarding technology and apps, the patients’ subjective perception of security with the disease through monitoring, adherence and sociodemographic factors.

Four distinct user categories were identified:

1. The Active Career Woman (Hannah)—Characterized by a dynamic, busy lifestyle and a low burden of disease, leading to little time and attention to health management.
2. The Curious Best Ager (Brigitte)—Typically motivated by a strong interest in exploring new technologies and a strong motivation for health management, due to a higher burden of disease.
3. The Routine-Oriented Soon-to-be Retiree (Klaus)—can be overwhelmed by new technologies, interest in self-management depending on the current burden of disease.
4. The Non-User—Often apprehensive about or unfamiliar with digital tools, with limited engagement in mHealth due to scepticism or lack of digital literacy.

**Figure 3.**
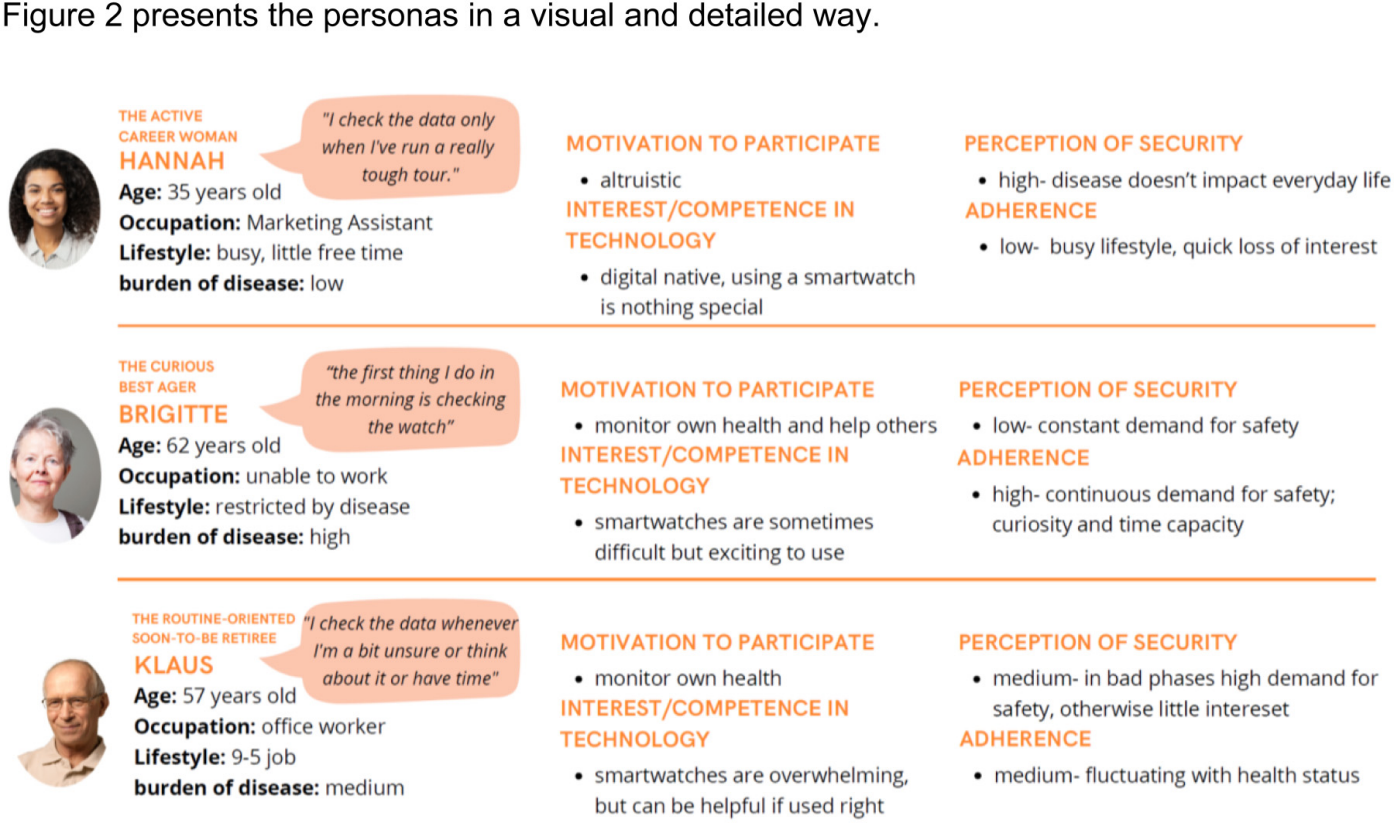
The figure visually details four distinct patient personas The Active Career Woman (Hannah), The Curious Best Ager (Brigitte), The Routine-Oriented Soon-to-be Retiree (Klaus), and The Non-User—which represent common types of CHF patients.

Figure 3 presents the personas in a visual and detailed way.

While the first three user categories are based on the different user types in the study, the fourth category is based on a type of user found in literature, characterized by having trust issues towards mHealth apps and a lack of technical literacy.


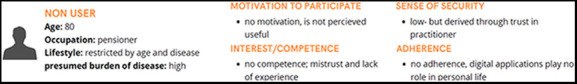


## Discussion

### Principal findings

The aim of this study was to investigate the impact of smartwatch self-monitoring on QoL in patients with CHF and their experiences with this monitoring method. The qualitative part of the study focused on patients’ usage patterns, attitudes towards smartwatch-based monitoring as well as perceived benefits and barriers. In addition, QoL questionnaires of participants were analyzed descriptively to further characterize the study population. Additionally, personas were developed to support HCPs’ decision-making in whom to offer a wearable. The four personas represent common types of CHF patients, their disease burden and willingness to use wearables.

Overall, CHF patients in this study spoke positively about self-monitoring with a smartwatch. They found it helpful and reassuring to be able to check their vital signs at any time. Accordingly, most patients reported using the watch and corresponding apps multiple times per day. Regular checking provided a general sense of reassurance, but some found the dependency burdensome. Benefits of using the watch included flexibility and simplification of disease management, increased awareness of health status, and the ability to compare vital signs over time.

According to the patients reports, functions or data they used or accessed most were heart rate data, oxygen saturation data, and the ECG. Yet, incorporating the new self-monitoring method in their day-to-day life was not trivial for every participant. Some perceived barriers such as lack of time, dependency on technical tools, and technical issues as burdensome. Other barriers that were mentioned included various errors in functionality, as well as the inability to use the app on their own smartphone and the associated effort of carrying two mobile phones.

The results of the quantitative analysis show that there may be a tendency for patients who participated in the interviews to have a higher QoL. However, the study population is too small to obtain statistically significant results.

Nonetheless, insights from the quantitative analysis of QoL were helpful in the development of the personas. This integration helped shape a more comprehensive understanding of patient profiles beyond their potential digital engagement and technological literacy. For the “Curious Best Ager,” higher disease burden and lower QoL may drive engagement with wearables to manage their condition, while the “Active Career Woman” with lower burden might perceive less urgency. The “Routine-Oriented Soon-to-be Retiree” illustrates how fluctuating QoL could lead to variable engagement, and for the “Non-User,” lower QoL or significant burden might contribute to the unwillingness to engage.

## Comparison to prior work

Although some wearables and remote monitoring in general have shown to positively impact outcomes such as hospital (re-)admissions^37^ and medication adherence,^38^ thus potentially improving care for patients with chronic diseases, potential challenges have to be weighed. Most commercially available smartwatches were not developed for medical use, but for supporting exercise or wellness^39^ and were validated with young and healthy users. Thus, the accuracy of used algorithms can be worse than expected. This could potentially lead to wrong conclusions in medical use cases.^39^ Hence, some authors call for the development of “medical watches” or devices specifically developed for patients with CHF.^40^ However, in our study, interviewed patients showed a clear preference towards a simpler technical set-up. They explicitly mentioned that wearing several watches and accompanying phones was burdensome (in our study, some patients continued to use their personally owned watch alongside the watch provided by the study team). They voiced a desire to collect their medical data on the same device they use in their everyday life. Thus, while a specific medical watch could provide more accurate data, patient acceptance and willingness to wear such an additional device is questionable.

Technical difficulties and in turn impaired ease of use were previously identified as relevant barriers against using mHealth tools for self-management in CHF^23^ and other chronic diseases.^30,41,42^ This barrier was also brought up in the interviews presented in this study, possibly having negative effects on adherence to the regular QoL assessments. This qualitative observation was reinforced by the analysis of the number of submitted questionnaires. Large variations were observed, as five users submitted only one questionnaire and four users submitted 14 or more questionnaires. We hypothesize that this variation is mostly explained by the experienced technical issues, since no interview participant rejected or opposed the general concept of submitting QoL questionnaires through the app. While improvements of the used tools could fix some of these issues, providing more support to users to deal with technical issues is necessary. Besides that patients with CHF are typically older and potentially less experienced with using technical tools, they also value support highly and are known to be more inclined to stop using a new technology when technical issues occur.^41,43^ In turn, the availability of a support infrastructure has been identified as a facilitator for usage of digital health tools.^44^ A 2024 study surveyed 49 patients with CHF on their online health information seeking behavior and discovered that information on management of CHF was a highly relevant area of interest for these patients.^45^ However, in the same study, 20% of participants were not aware of the potential of wearables to support CHF self-management. Participants also had the impression that wearables were only used by highly active people in a fitness context and thus did not see these tools as an option for them.^45^ This is in contrast to our study, in which participants with previous experience with wearables were able to cite several benefits for their disease management through this tool. These contrasting findings could indicate that while patients with CHF are looking for tools to improve their self-management, a significant part of these patients is not aware of the potential for support that wearables could provide. This strengthens the observation that patients with CHF need support infrastructures and HCP to be both introduced to wearables and to have someone to contact when usability problems arise. The results from our work strengthen the need for future research to develop and evaluate ideal infrastructures for technical support and implementation strategies for introducing wearables and mHealth, especially for older patients.

For HCP aiming to provide both patient-centered and (cost-)effective care, it can be difficult to decide whom best to offer a wearable for self-management support. Based on the results of this study and on literature research, four personas were developed. The personas represent common types of CHF patients, their disease burden and willingness to use wearables: the “active career woman,” the “curious best ager,” the “routine-oriented soon-to-be retiree,” and the “non-user.” Personas have been used before in various studies investigating the adoption of mHealth.^31–33^ Regarding patients with CHF, Holden et al.^46^ in 2016 developed six personas to be used by designers. Although these personas are aimed at a different target group, their personas align well with the personas presented in this study—for example, their persona “Barbara” shares important traits with the “curious best ager” in our study. Their persona “Frederick” can be compared to our “Non-User,” as both have no intention to use digital health tools. However, our work also includes the perspective of younger users, exemplified in the persona of the “active career woman.” In addition, it is plausible that the intention to use wearables differs from adoption of digital health tools in general, warranting the extension of existing personas to reflect these recent new tools. Furthermore, our personas include the aspect of sense of security, which was identified as relevant through qualitative interviews and, to the authors’ knowledge, has not been described before.

## Limitations

Although the disease-specific KCCQ-12 was used to survey QoL, our study deviates from the usual two-week recall period, limiting comparability to other studies in the cohort of patients with CHF. In addition, a high rate of missing data was observed. Still, the questionnaire data provides valuable insights and reflects the development of QoL over the course of a chronic disease.

For the interviews, 12 patients were successfully recruited. In comparison to purely quantitative studies, this appears as a small sample. However, for a qualitative, phenomenological approach within a homogenous group of participants, a smaller sample size can be appropriate and effective to reach both thematic and coding saturation,^35^ which was achieved in our study. Key themes—such as technical difficulties, the inability to use the study device for private purposes, improved self-monitoring, and an increased sense of security—were mentioned repeatedly across interviews. Furthermore, all participants consistently expressed a positive attitude toward self-monitoring. This homogeneity in the interview data allowed us to conclude that theoretical saturation was reached after 12 interviews. Yet, the risk of selection bias needs to be weighed. It is possible that only patients with a higher openness or previous positive experiences with new technologies agreed to participate in the study.

Although both qualitative and quantitative data were collected, integration was not possible due to data privacy regulations, hindering the possibility to gain an even deeper understanding and generate further insights into the studied phenomena. Future research should aim to ensure that a chosen study design, such as mixed-methods, can be performed while still following data privacy laws.

Furthermore, all patients included in the study are less than 60 years old, which is not representative for the largely elderly cohort of patients with CHF.^5^ This could have led to more optimistic reports and limited generalizability of the presented results to the large and typically older group of CHF patients. While this is a known problem in digital health research,^47,48^ future research should strategically aim to include representatives from older age groups in studies and clinical trials. To this end, some promising strategies have been developed, such as offering in-person informed consent sessions with the opportunity to ask questions face-to-face and to help with setting up the studied technology,^49^ broadening eligibility criteria that disproportionately exclude elderly patients (e.g. use of hearing or visual aids, multiple comorbidities),^50^ or enabling modifications in the studied technology such as a larger font.^51^

At the same time, patients in this study reported technical issues with the app used for data collection. These reports and complaints took over large parts of the interviews and could have both overshadowed positive experiences and biased the results of the KCCQ-12 as the technical issues made it difficult to submit completed KCCQ-12 forms. Regarding health status of the patients in the study presented here, our cohort reported a lower KCCQ-12 score compared to a recent meta-analysis (52.5 vs 60.9^7^). However, both these scores fall into the same category of KCCQ-12 (“fair to good QoL,”^31^), indicating that our cohort is in line with the expected health status of CHF patients according to the meta-analysis of Moradi et al.^7^

In addition, it has to be considered that the presented results were produced in a regulated and fully funded study context. Implementation of a wearable and mHealth app in regular care settings without the support infrastructure of a clinical trial could lead to unforeseen issues that were not discovered in our study, including financial barriers. Although financial barriers to using a wearable or mHealth app were not mentioned by the study participants, it is important to note that the German statutory health system has created a financing scheme for reimbursing patients to use mHealth apps that collect subscription or other fees as well as wearables. Generalizability to other settings should be assessed carefully, although several other countries are considering adopting a similar system to facilitate the use of digital health applications.^52,53^

## Conclusion

Overall, CHF patients in this study spoke positively about self-monitoring with a smartwatch and perceived the regular QoL assessments through the app as feasible. They found it helpful and reassuring to be able to check their vital signs at any time. Most patients reported using the watch and corresponding apps multiple times per day. Regular checking provided a general sense of reassurance, but some found the dependency burdensome. Benefits of using the watch included flexibility and simplification of disease management, as well as increased awareness of health status and the ability to compare vital signs over time.

Based on these insights, four personas were developed to reflect common patient types with differing levels of disease burden and digital engagement. These personas can support healthcare professionals in identifying patients who may benefit most from wearable-based monitoring. Looking ahead, integrating such tools into clinical workflows—for example, for tailored follow-up or remote monitoring—could enhance patient-centered CHF care and should be explored in future research.

## Supplemental Material

sj-pdf-1-dhj-10.1177_20552076251375967 - Supplemental material for Integrating wearable mobile health technologies into chronic heart failure management: Insights from a mixed-methods study and persona development Supplemental material, sj-pdf-1-dhj-10.1177_20552076251375967 for Integrating wearable mobile health technologies into chronic heart failure management: Insights from a mixed-methods study and persona development by Laura Svensson, Carolin Anders, Christoph Dieterich, Oliver Heinze, Petra Knaup and Lina Weinert in DIGITAL HEALTH

sj-pdf-2-dhj-10.1177_20552076251375967 - Supplemental material for Integrating wearable mobile health technologies into chronic heart failure management: Insights from a mixed-methods study and persona development Supplemental material, sj-pdf-2-dhj-10.1177_20552076251375967 for Integrating wearable mobile health technologies into chronic heart failure management: Insights from a mixed-methods study and persona development by Laura Svensson, Carolin Anders, Christoph Dieterich, Oliver Heinze, Petra Knaup and Lina Weinert in DIGITAL HEALTH

sj-pdf-3-dhj-10.1177_20552076251375967 - Supplemental material for Integrating wearable mobile health technologies into chronic heart failure management: Insights from a mixed-methods study and persona development Supplemental material, sj-pdf-3-dhj-10.1177_20552076251375967 for Integrating wearable mobile health technologies into chronic heart failure management: Insights from a mixed-methods study and persona development by Laura Svensson, Carolin Anders, Christoph Dieterich, Oliver Heinze, Petra Knaup and Lina Weinert in DIGITAL HEALTH
