# Supplementary material for: Integrating wearable mobile health technologies into chronic heart failure management: Insights from a mixed-methods study and persona development
Source: Digit Health. 2025 Oct 9;11:20552076251375967. doi: 10.1177/20552076251375967 (PMC12515331; doi:10.1177/20552076251375967)
Supplement: sj-pdf-1-dhj-10.1177_20552076251375967 - Supplemental material for Integrating wearable mobile health technologies into chronic heart failure management: Insights from a mixed-methods study and persona development [file sj-pdf-1-dhj-10.1177_20552076251375967.pdf]

Interview Guide: Feasibility and experiences with self-monitoring

Information about data protection

[General information on the topic of the interview, audio recording, anonymisation, data protection, …]

Do you have any questions up to this point? *Start recording*

Introduction

Opening question:
As part of our study, you are using a smartwatch and the phellow app to record your vital data, such as your heart rate. What did you think of the idea when you first heard about it?

Optional:
If you think back to the beginning of use,
What expectations did you have of the devices you received from us? And to what extent were these expectations met?

Please briefly try to remember the time before the study. How was your interest in self-monitoring your vital signs such as heart rate, blood pressure, and weight?

Optional:
If very interested: How have you monitored your vital signs such as heart rate, blood pressure, weight yourself so far? What tools have you used? To what extent have you also considered a smartwatch for this?

Experience with the use of wearable (+ smartphone)

We are particularly interested in your experiences with the devices you have received from us. How have you got on with the devices so far?

To what extent did you have all the necessary information that was important for use?
How often do you wear the smartwatch (and why)?
How often do you use the phellow app and for what?

Optional:
How often do you fill out questionnaires within the app?
How often do you enter self-measured values such as blood pressure or weight into the app?

Optional (for infrequent use):
What prevented you from using the app more often?

**To what extent have you experienced technical difficulties?**<br>Optional: What did you do in case of technical difficulties?	
**Experience with self-monitoring**	
**In the next questions we are interested in how you deal with the measured data.**<br><br>**Do you also look at your measurement data?**<br>Optional:<br>If yes, where do you view your data? (mobile phone or app)<br>In which situations do you view your data?<br>Has anything changed over the course of your use?<br>If not, what are the reasons?<br><br>**When you look at your readings, how do you deal with the information?**<br>Optional:<br>How do you react when your values are too high or too low?<br>How do you feel then?<br>To what extent do you change your behaviour?<br><br>**You can use the smartwatch and the app to measure and monitor your vital data in everyday life. Do you perceive this as an enrichment or a burden and why?**<br><br>**How has your approach to the disease changed since you started using the smartwatch (and the phellow app)?**<br>Optional:<br>What other effects have occurred as a result of being able to view your measured values yourself?	
**Implementability and willingness to donate data**	
**What else should the app be able to do to offer you even more added value?**<br><br>**Think back to the time when you decided to take part in the study.**<br>**What motivated you to take part in the study?**<br><br>**By participating in the study, you are also making your data available to us, which is necessary for the research.**	

**To what extent is this aspect important to you?**<br>Optional: To what extent were you aware that you were making your data available to research?<br><br>**To what extent are you willing to make your measured values available to research beyond the scope of the study?**	
**Conclusion**	
**What other thoughts would you like to share with me?**	
**Thank you very much for the open discussion and your comments.**	
