# Supplementary material for: Integrating wearable mobile health technologies into chronic heart failure management: Insights from a mixed-methods study and persona development
Source: Digit Health. 2025 Oct 9;11:20552076251375967. doi: 10.1177/20552076251375967 (PMC12515331; doi:10.1177/20552076251375967)
Supplement: sj-pdf-2-dhj-10.1177_20552076251375967 - Supplemental material for Integrating wearable mobile health technologies into chronic heart failure management: Insights from a mixed-methods study and persona development [file sj-pdf-2-dhj-10.1177_20552076251375967.pdf]

Code System – Kardiostudie

**Code**	**Explanation**	**Anchor quote**
**Self monitoring**		
Usage		
Usage patterns	Statements about how often and in what way patients use the watch for self-monitoring	*Actually, I look at them every day, so I always check how things have been today and, as I said, if I'm not feeling well or something, then I also look at how they are at the moment, the data. (Interview 8, Para 39)*
Usage development	Statements on how user behavior has developed over the period of use	*Yes, I'm looking at more and more things, now I'm using the ECG, which I didn't try at the beginning, but I've tried it now. (Interview 4, Para 47)*
Attitudes towards self-monitoring		*I notice how my body makes me feel in the end and I don't stress myself with the vital signs, I don't want to put myself under pressure. (Interview 10, Para 47)*
Before the Study		
Previous behavior patterns	Behavior patterns related to self-monitoring that patients had before the study	*Before the study there was only blood pressure measurement and nothing else, so no further options. (Interview 9, Para 11)*
Expectations		
with other measuring methods	Patients' previous experience with measurement methods other than a smartwatch	*We have a scale from Medisana, I think, which uses Bluetooth to automatically enter the weight into an app called Vitadoc after weighing. (Interview 5, Para 10)*
with smartwatch	Patients' previous experience with a smartwatch for self-monitoring	*I once got a smartwatch myself [...] just a normal one and I still have it at home and tested it myself, it only shows wrong values. (Interview 9, Para 5)*
Attitudes	Patient attitudes towards self-monitoring with a smartwatch before the study	*I thought it was a good idea, so I think this whole digitalization should be brought forward a bit anyway, so I think it's a great idea. (Interview 11, Para 3)*
Benefits of self-monitoring	Patient statements on the advantages of using a smartwatch for self-monitoring	
Flexibility and simplification		*The watch really helps me a lot because it measures constantly and I don't have to run to my blood pressure device or anything, I have to say that the watch is worth its weight in gold. (Interview 9, Para 105)*

Comparison of vital data		*Yes it will be easier for me to know my blood pressure weight and heart rate from previous dates. (Interview 1, Para 10)*
Facilitates contact with physicians		*[...] I could turn to a doctor with a better feeling, that I have something to show. (Interview 11, Para 37)*
Overview of health status		*I think it's actually quite good that it's also useful for you to reflect on it again how up-to-date your blood pressure is, for example, or to check all the data yourself. (Interview 5, Para 6)*
Increased sense of security		*Even if I notice, oh I think I am trembling, it is not yet atrial fibrillation but better calm down now. I just see that and before I couldn't, so I feel safer. (Interview 6, Para 13)*
Effects of self-monitoring	Patient statements on the effects of using a smartwatch on their self-monitoring behavior	
Individual responsibility		*So with this research it makes me more responsible in monitoring my blood pressure and everything. (Interview 1, Para 18)*
Effects on dealing with disease		*I don't know whether the Apple Watch has really changed the way I deal with my illness. (Interview 10, Para 47)*
increased awareness of health status		*I consciously look at them on days or after days when I've either done something particularly strenuous or something like that or when I realize I'm not feeling well.(Interview 6, Para 89)*
Controlling the health status		*I look at the data everyday, I always check how I have been today and, as I said, if I'm not feeling well or something, I also look at what the data says at the moment. (Interview 8, Para 39)*
Dealing with deviating values		
Reactions	Patients' internal reactions to deviating values that are shown to them by the smartwatch	*So and yes, I can't do anything then, so I'm very agitated inside, very restless, yes, maybe it's also too much imagination, what's going on there, yes, fears come up about what could be or why it's like this now […]. (Interview 3, Para 78)*
Strategies	Concrete strategies of patients in the event of deviating values that are shown to them by the smartwatch	*I then simply try to avoid this strain or to do it in such a way that I can ultimately say I can manage it so that my pulse doesn't go up. At work, I just do the heavy work at a slower pace. I then adjust my pace and then I can keep my heart rate down. (Interview 12, Para 39)*

**Expectations**	Statements on the patients' expectations on the study	
Stress through self-monitoring	Patient statements that self-monitoring could cause stress	*I was afraid it would be a burden because I really thought at the beginning I look at it all the time and oh dear oh dear. (Interview 6, Para 99)*
Data protection	Patient statements on expectations regarding data protection	*[…] It was known that this would be anonymized and it's not for my benefit but for the benefit of everyone. (Interview 12, Para 5)*
Functional scope	Patients' expectations of the functional scope of smartwatches	*At first I was expecting an atrial fibrillation message, but then I discussed and read it again and then it was clear that this was not the case, but that I could check it myself. (Interview 6, Para 7)*
Personal use of the devices	Patients' expectations regarding the personal usage of study devices	*Well, I was hoping that I would be able to use it more [to manage my personal life] but that's just not possible. (Interview 7, Para 15)*
Simplify monitoring	Patient statements that the devices simplify self-monitoring	*My expectations have only been fulfilled in that I now have [...] functions that I didn't have before, such as ECG and that I can measure my pulse and oxygen level at any time. (Interview 4, Para 14)*
**Suggestions for improvement/ factors to increase acceptance**		
Extension of functional scope	functions patients would wish for	
Alarm in the event of anomalies		*Maybe if something is noticeable that then I don't know if that's also in there, that then an alarm comes or a push message like you have heart stumbling or something was irregular here, that you somehow get an info there. (Interview 11, Para 49)*
Visualisation of data		*It will also help if it would also show what was been saved or what's been stored, it will only say in the history that I made I stored the data on this day but not the data itself. (Interview 1, Para 71)*
Reminder for medication		*[…] there could be e reminder when I have to take my medication, i.e. at what times or at what time of day. (Interview 3, Para 85)*
Blood pressure measurement function		*Of course, it would be nice if you could measure blood pressure with this device. (Interview 4, Para 24)*
Login status	Proposal to customize the login status display	*I have uploaded my pulse, heart rate and blood pressure data every day, but I can't see that I'm no longer logged in. (Interview 2, Para 15)*
Provision of user manual	Proposal for the provision of a user manual	*Yes, a simple guide [...]. Because I still haven't really got the hang of it yet. (Interview 4, Para 32)*

Medical monitoring of the values	Suggestion to have the collected values monitored by a doctor	*I think it would also add value for my practitioner or something like that if you could say hey, you could download a PDF or something like that. (Interview 10, Para 53)*
Use of personal devices	Proposal to enable the use of personal devices for self-monitoring	*I asked in advance if the app could be transferred to my personal device because I am already equipped with an Iphone, I am equipped with an Apple Watch so to do that twice would have been unfavorable. (Interview 2, Para 2)*
Flexibility in data collection	Proposal to allow flexibility in the collection of data	*That would be cool if you could just say I'll add something again because sometimes when I'm distracted I'm just a person who always enters it very late before I go to bed and then it's sometimes a bit critical with the time (Interview 10, Para 29)*
Motivation to participate in the study		
interest in monitoring the own health status	Statements on own interest in self-monitoring as motivation to participate in the study	*Yes, because I had heart failure [...] it was important for me to look at my values during exercise and also to check how high my heart rate is in different situations in order to find out where I could perhaps improve myself and where I might need to slow down a bit. (Interview 10, Para 9)*
Helping other patients	Statements on the desire to help other patients as motivation to participate in the study	*I can then help other people, so by taking part, it will help someone else at some point, so I think that's good, too. (Interview 11, Para 61)*
Curiosity about technology	Statements on own curiosity about technology as motivation to participate in the study	*[..] that helped me a lot to understand, do I need such a watch, do I not need it and how do I use it? (Interview 6, Para 5)*
More attention for heart failure patients	Statements on the desire to generate more attention for heart failure as motivation to participate in the study	*[..] that at some point you might really say ah yes, these heart patients need more attention, what do we have at the moment, nothing.(Interview 9, Para 122)*
Improve own situation	Statements on the desire to improve one's own situation by participating in the study	*To also pay a little attention to how the values are and to be a little more aware of it and see how it works. (Interview 5, Para 60)*
Supporting research/gaining knowledge	Aussagen zum Wunsch die Forschung durch Studienteilnahme zu unterstützen und Erkenntnisgewinn zu fördern	*[…] because there are many who also have the disease and who can then later benefit from the data that I may not now, but others will have success later and you can fight it sooner and better. (Interview 12, Para 55)*

**Barriers**		
Lack of time	Statements on lack of time as an obstacle to using the watch for self-monitoring	*I went back to work starting in June, so maybe that’s the reason why I look at it lesser than before when I was at home cause at work I always I think about work than checking it. (Interview 1, Para 57)*
Internet connection required	Statements on difficulties with the Internet connection as an obstacle to using the watch for self-monitoring	*[…] I always have to look where I can get wifi and then where I can transmit the data, so it's a bit annoying. (Interview 4, Para 14)*
No medical monitoring of the values	Statements that the values collected in the study are not monitored as a barrier to regular use	*[…] because I'm not connected to the medical field, I'm connected to the computer center that tests it [...]I don't have any medical use yet. (Interview 4, Para 12)*
Little insight into data	Statements that the limited access to the data hinders regular use	*The data is extracted and then ends up somewhere, but I can't look at it in such detail. (Interview 11, Para 47)*
Dependence on devices	Statements that dependence on the devices is perceived as a barrier with self-monitoring	*But yes I always have to have a bag with me and have to take the phone with me and the data gets transferred. (Interview 4, Para 16)*
Wearing comfort	Statements on wearing comfort that hinder the motivation for self-monitoring	*For example, I find wearing the watch at night sometimes exhausting because it somehow often squeezes my hand a bit. (Interview 10, Para 45)*
functional scope	Statements on functional scope that hinder self-monitoring	*I thought the watch would also measure blood pressure and a bit more stuff >laughs< and you could do a bit more with your mobile phone. (Interview 8, Para 7)*
Technical problems	Statements on technical problems that have hindered the use of the watch for self-monitoring	*It often updates itself and sometimes it really does freeze. (Interview 10, Para 19)*
Appointments not up to date		*I have now seen that it has also entered all the appointments I have at the university hospital, so not all of them, but somehow some of them are sometimes entered if I have them changed due to appointments that make it impossible for me to come by, then the appointment in the app does not change. (Interview 10, Para 19)*
Data transmission		*Yes, so at the beginning of the use I entered my data, clicked on send and then it said 35 vital data could not be transmitted every time, so I was a bit*

		unsure whether anything was transmitted at all because the display was always on could not be transmitted. (Interview 10, Para 21)
Low battery life		*You always have to remember to charge it, that's just >laughs< [...] it's often happened that I've been out and about and the smartwatch was empty and then of course it didn't measure anything anymore. (Interview 8, Para 33)*
No personal use of study device	Statements that personal devices can't be used for the study as a barrier for self-monitoring	*Well, I was hoping that I would be able to use it more in my personal life, but that's just not possible. (Interview 7, Para 15)*
Missing manual	Statements on the lack of a manual that hindered the use of the devices	*[…] I didn't know what to do at first. (Interview 5, Para 16)*
Usability	Statements on usability that hindered the use of the devices	*It took me some time to work it out, at the beginning I said at first I said oh don't adjust anything and then I said yes let's see so the watch works but I say I just can't make friends with the Iphone >laughs<. (Interview 9, Para 55)*
**Acceptance/contributing factors**		
Financial support	Patient statements on how financial support could increase the acceptance of smartwatches for self-monitoring	*I think it's great when you already have these technical possibilities that you can almost afford it or maybe even the health insurance companies will take pity on you and give you a subsidy at some point. (Interview 6, Para 129)*
Fun in handling	Patient statements on the extent to which having fun with the Watch contributes to acceptance of the device	*It makes me curious every time and also it makes me happy to know […] if its good or to know if its bad. (Interview 1, Para 63)*
Technical reliability	Patients' statements on the extent to which technical reliability increases their acceptance of the devices	*[…] so in the meantime you rely on it a lot when you realise it's really accurate and then you say you'll make the effort. (Interview 9, Para 71)*
Social recognition	Patients' statements on the extent to which social recognition increases their acceptance of the devices	*And my daughter knew all about it >laughs< she was very envious. (Interview 7, Para 33)*
Useful functions	Patient statements on the extent to which useful functions of the watch increase their acceptance	

Training function of the watch		*I also regularly check the fitness app with the training rings to see how things have developed or when a ring wasn't closed. (Interview 5, Para 40)*
Pulse		*[…] that I can measure my pulse and oxygen levels at any time. (Interview 4, Para 14)*
ECG		*As well as writing the ECG, which I think is great […]. (Interview 9, Para 116)*
Sleep mode		*I think it's great that you can really darken it in the evening in sleep mode. (Interview 7, Para 97)*
Blood oxygen		*What I find important is this blood oxygen meter [...], so I find that important for us heart patients. (Interview 9, Para 116)*
Mindfulness function		*For example, I always find this mindfulness exercise really exciting >laughs< [...] at some point I just go like this, now breathe for a minute, and I think that's really great, where I think that's what I do all day, but then I'm really focussed and I'd like […] to do it more often (Interview 7, Para 97)*
Personalisation options	Patient statements that personalisation options of the watch increase their acceptance of the devices	*The use is actually simple and well explained I just switched off the sound because it actually annoys me when I am chatted to by the watch every day. (Interview 6, Para 13)*
Previous experience with devices	Patients' statements on the extent to which previous experience with smartwatches increases their acceptance of the devices	*I have an Apple Watch and an iPhone myself and know my way around a bit and yes, it wasn't a problem. (Interview 5, Para 8)*
Simple operability	Patients' statements on the extent to which the ease of use of the Watch increases their acceptance of the devices	*[…] I mean, that's all relatively self-explanatory. (Interview 3, Para 30)*
